# Clarification Added to Methods

**DOI:** 10.1001/jamanetworkopen.2023.34513

**Published:** 2023-09-12

In the Original Investigation titled “Workforce Attrition Among Male and Female Physicians Working in US Academic Hospitals, 2014-2019,”^1^ published July 17, 2023, the following sentence was added to the Methods section to clarify how physician gender was assessed: The Centers for Medicare & Medicaid Services database uses the term *gender* for the characteristic and the values as “M/F/U.” This article has been edited.^1^
